# Overlap Extension Barcoding for the Next Generation Sequencing and Genotyping of Plasmodium falciparum in Individual Patients in Western Kenya

**DOI:** 10.1038/srep41108

**Published:** 2017-01-24

**Authors:** Brandt Levitt, Andrew Obala, Scott Langdon, David Corcoran, Wendy Prudhomme O’Meara, Steve M. Taylor

**Affiliations:** 1 Department of Molecular Genetics and Microbiology, Duke University Medical Center, Durham, NC, USA; 2 Moi University School of Medicine, College of Health Sciences, Nandi Rd, Eldoret, Kenya; 3 Duke University DNA Analysis Facility, Department of Immunology, Duke University Medical Center, Durham, NC, USA; 4 Duke Center for Genomic and Computational Biology, Duke University Medical Center, Durham NC, USA; 5 Division of Infectious Diseases and Duke Global Health Institute, Duke University Medical Center, Durham, NC, USA

## Abstract

Large-scale molecular epidemiologic studies of *Plasmodium falciparum* parasites have provided insights into parasite biology and transmission, can identify the spread of drug resistance, and are useful in assessing vaccine targets. The polyclonal nature infections in high transmission settings is problematic for traditional genotyping approaches. Next-generation sequencing (NGS) approaches to parasite genotyping allow sensitive detection of minority variants, disaggregation of complex parasite mixtures, and scalable processing of large samples sets. Therefore, we designed, validated, and applied to field parasites an approach that leverages sequencing of individually barcoded samples in a multiplex manner. We utilize variant barcodes, invariant linker sequences and modular template-specific primers to allow for the simultaneous generation of high-dimensional sequencing data of multiple gene targets. This modularity permits a cost-effective and reproducible way to query many genes at once. In mixtures of reference parasite genomes, we quantitatively detected unique haplotypes comprising as little as 2% of a polyclonal infection. We applied this genotyping approach to field-collected parasites collected in Western Kenya in order to simultaneously obtain parasites genotypes at three unlinked loci. In summary, we present a rapid, scalable, and flexible method for genotyping individual parasites that enables molecular epidemiologic studies of parasite evolution, population structure and transmission.

Malaria kills over 400,000 people annually throughout the tropics^1^, and new tools are continually needed to track and type parasites to improve disease control. *Plasmodium falciparum*, the deadliest and most virulent malaria parasite species, has a large and highly diverse genome^2^. Population genetics offers a research toolset to elucidate the parasite’s transmission patterns and define and track medically-important genotypes such as those responsible for more severe disease or conferring drug- or vaccine-resistance^3^,^4^,^5^. Recent advances in next generation sequencing (NGS) technology have broadened the utility of these approaches^6^, allowing more comprehensive detection of parasite genotypes present in complex parasitemias^7^. In doing so, these approaches can enable better understanding of minority variants including their transmission^8^, contribution to parasite population structure, and involvement in drug and vaccine candidate resistance^9^.

Most common approaches to parasite genotyping are not easily scalable and therefore poorly suited to large molecular epidemiologic studies. These common approaches include PCR amplification followed by restriction fragment length or sequence-specific oligonucleotide polymorphism analysis, direct Sanger sequencing, or capillary electrophoresis for short-tandem repeat multilocus typing^10^,^11^,^12^,^13^,^14^,^15^. In addition, the utility of these approaches is undermined by the polyclonality common to high transmission regions such as Sub-Saharan Africa due to diversity at the same nucleotides in multiple parasites within individual patients^14^,^16^. More recent approaches use NGS platforms to reconstruct whole genomes, but this approach is complicated by the parasite’s genomic architecture, polyclonality of parasitemias, and the need for sophisticated genome-scale assembly and analytic tools^6^,^17^,^18^. NGS platforms have also been employed in ecological studies to sequence amplicons generated from pools of parasites^19^,^20^, but heretofore technical constraints have limited the ability to assign sequence reads to individual input parasites. Generating high-dimensional parasite genotypes from individual parasitemias would enable new approaches to scalable, rapid, and manageable genotyping tools for both parasite population surveillance and patient-level analyses of genotype-phenotype correlations.

In this study, we designed, validated, and applied an overlap extension barcoding and multiplexing protocol followed by NGS that permits the rapid and parallel sequencing of multiple polyclonal parasite gene targets in individual infections. Using this approach, we simultaneously genotype *P. falciparum* population genetic markers for population structure, drug resistance, and vaccine-resistance in both control and field-collected DNA samples from Western Kenya. This modular, multiplex and quantitative approach to *P. falciparum* genotyping allows for estimation of allele frequencies and haplotypes in multiple gene targets within complex polyclonal *P. falciparum* parasitemias. Our approach has the potential to improve both routine surveillance and research studies.

## Methods

### Preparation of control DNA mixtures

Control DNA mixtures were prepared from genomic DNA (gDNA) extracted from reference strains of *P. falciparum* acquired from the Malaria Research and Reference Reagent Resource Center (BEI Resources). Six mixtures of MRA-102G (3D7), MRA-150G (Dd2), MRA-152G (7g8), MRA-155G (HB3), MRA-731G (FCR3-FMG), and MRA-159G (K1) at 500 ng in 50 microliters of TE were prepared in proportions as described in Table 1A and used as template in subsequent PCR amplifications.

### Multiplex amplification and barcoding

**Figure 1:**
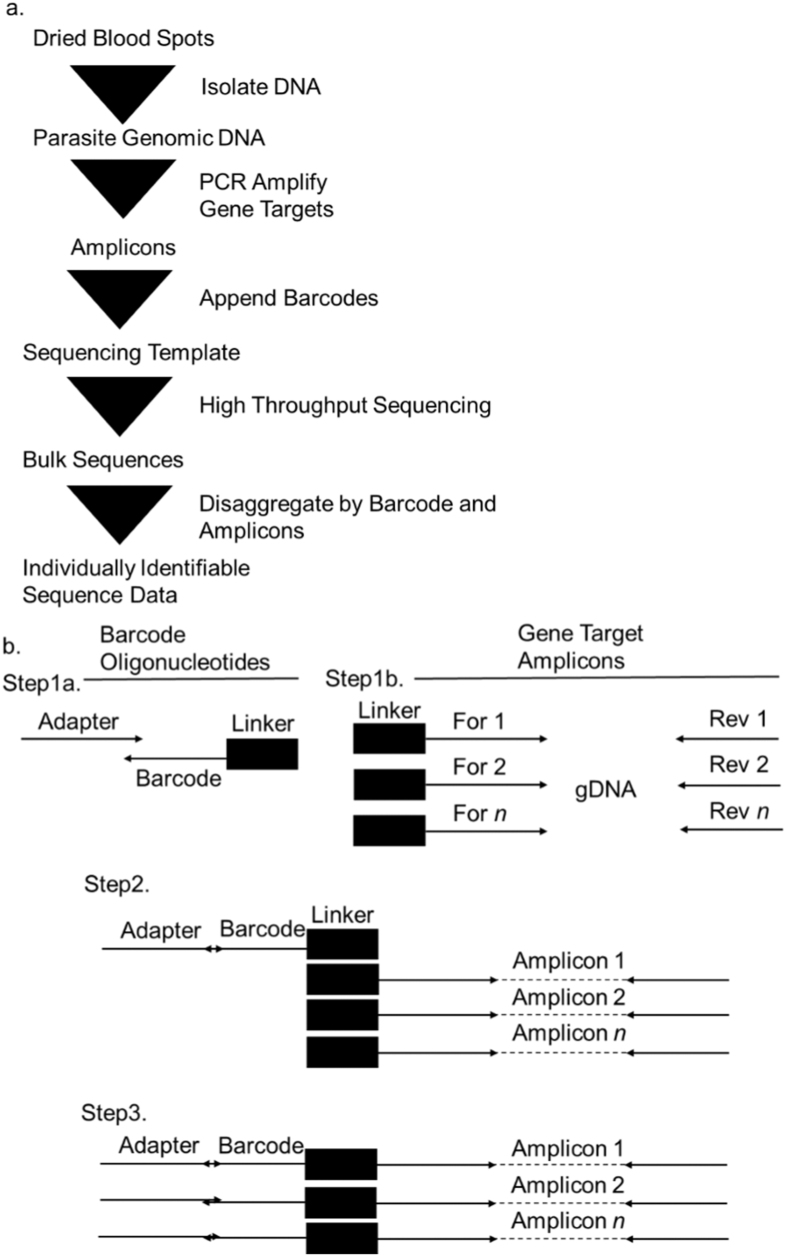
(**a**) Workflow for preparation of individually identifiable sequence data from dried blood spots. (**b**) PCR Schema to append unique barcode oligonucleotides to multiplexed gene-target amplicons.

We used the PCR schema outlined in Fig. 1 in order to generate barcoded amplicons of three gene targets that included adaptor sequences necessary to sequence on an Ion Torrent platform. Two gene targets, *pf-csp* and *pf-ama1*, were selected as markers for parasite strain typing owing to their polymorphic nature and lack of indels^21^,^22^,^23^,^24^,^25^. A third gene target, *pf-k13*, was selected because known polymorphisms confer artemisinin resistance^4^. Primers were designed by Primer3 (www.primer3.org) to 1) amplify polymorphic segments of each target, 2) have Tm values at 55C, and 3) generate similarly-sized amplicons of 321 bp (*pf-csp*), 305 bp (*pf-ama1*), and 306 bp (*pf-k13*), in order to mitigate bias in sequencing towards shorter amplicons.

First, we generated “barcode oligonucleotides” by Taq extension of primers containing the IonTorrent Adaptor A and each barcode followed by a linker (Fig. 1 Step 1A). This reaction consisted of 200 nM barcode primers, 200 nM IonTorrent Adaptor A forward adapter, 1X Taq buffer, 200 μM deoxynucleotides, 1.5 mM magnesium chloride and 0.5 units of Invitrogen platinum Taq (Life Technologies, BioRad); this reaction was incubated for 15 minutes at 95C, and then underwent 25 cycles of 95C for 30 s, 60C for 30 s, and 72C for 1 m, followed by 5 m at 72C. The 42–44 bp IonTorrent Adaptor A reverse primers contained 3 parts 1) the first 14 nucleotides were the reverse complement of the linker, 2) the next 10–12 base pairs comprised 192 unique barcodes, and 3) the remaining 18 nucleotides were the reverse complement of IonTorrent Adaptor A (Table S3). Each of the 192 unique IonTorrent Adaptor A reverse primers (IonTorrent Adaptor A reverse barcode 001–192) was added to a unique well of two 96 well plates while the common IonTorrent Adaptor A forward primer was added to every well.

In parallel with this, we generated amplicons of *pf-csp, pf-ama1*, and *pf-k13* by multiplex PCR of *P. falciparum* gDNA for each individual (Fig. 1 Step 1B). For each target, forward amplicon primers began with a linker sequence which facilitated the joining of the parasite amplicons to barcodes. Reverse primers ended with an IonTorrent adaptor sequence which is required for bead templating during sequencing. To generate the parasite amplicons, reactions included 1 uL of parasite gDNA, 200 nM final concentration each of the forward Linker primers for each gene target and reverse TrP1 primers, and enzymes as above for a final reaction volume of 25 uL; cycling was identical to above. The product of this reaction was a mixture of amplicons of each of the three gene targets containing a linker at the 5′ end and the TrP1 adaptor at the 3′ end.

Finally, we generated full length sequencing libraries from the Fig. 1 Step 1A and Step 1B (Fig. 1 Step 2). The final set of primers were designed to amplify the full length library consisting of the barcode oligonucleotides (IonTorrent Adaptor A-Barcode-Linker) and the gene target amplicons (Linker-Amplicon-TrP1) using the products of Fig. 1 Step 1A and Step 1B as template. To generate these full length sequencing libraries, reaction templates consisted of 2.5 uL each of barcode oligonucleotide and gene target amplicons; this mixture served as template, which was amplified by overlap extension PCR using mastermix without primers for the first 10 cycles and adding adaptor primers (IonTorrent A and TrP1) at 200 nM each for the remaining 15 cycles. (Fig. 1 Step 3).

These amplification products from all reactions were pooled in equal volumes, purified by ethanol precipitation, and electrophoresed on a 1% Tris Borate EDTA agarose gel, from which bands at 370 bp, 371 and 384 bp were excised and purified together using a Qiaquick Gel purification kit according to manufacturer instructions (Qiagen, Valencia, CA, USA). The final product was analyzed by Tapestation to ensure expected fragment size and then used as template for IonTorrent sequencing using a single 318 chip.

### DNA extraction from field parasites

We used *P. falciparum* parasites collected from 180 participants in a cross-sectional study of parasite prevalence in Webuye, Kenya, conducted between May 2013 and July 2014^26^. 177 of the 180 participants tested positive for *P. falciparum* parasites using a commercial rapid diagnostic test (RDT) targeting the *P. falciparum* antigen HRP2 (Standard Diagnostics SD Bioline Malaria Ag Pf (HRPII)). The remaining 3 participants that tested negative by RDT were false negatives and contained *P. falciparum* gDNA. From each person, fingerprick blood was collected as a dried blood spot (DBS) onto Whatman filter paper and stored in individual plastic bags with desiccant. From each DBS a 5 mm hole punch was collected, and gDNA was isolated using a Chelex-100 protocol^27^.

### Allele variant analysis

Raw sequencing reads output as a FASTQ file were first trimmed of sequencing adaptors, filtered to remove reads of less than 150 bases using FastX toolkit, and then partitioned by unique barcode using the FastX barcode splitter. We allowed reads lacking the first 2 nucleotides of the 11 mer barcode sequence, but censored reads that harbored other deletions or substitutions in the barcode sequence. Barcodes and linker sequences were then trimmed from the 5′ end of reads using the FastX toolkit trimmer program, and all reads were mapped to reference sequences for *pf-csp* (GenBank: XM_001351086.1), *pf-ama1* (XM_001347979.1), or *pf-k13* (XM_001350122.1) using BWA-MEM with parameters to favor the mapping of high quality reads with allowances for indels and mismatches, including a minimum seed length of 19, minimum quality score to output of 30, and mismatch penalties of 4 and indel penalties of 6^28^. Mapped reads were input to Samtools MPILEUP to calculate the number of reads containing reference or alternate nucleotides at each position in the aligned reads. Within mapped reads, we censored individual nucleotides with quality score <25 as well as indels, owing to the expected absence of these in the gene targets. Allele frequencies were defined as the proportion of all mapped reads at a locus that harbored a reference or mutant allele.

### Haplotype inference analysis

Quality-filtered reads were mapped to the reference sequences using the map2 algorithm from the TMAP alignment suite (default parameters). The alignment result for each read was processed to remove any homopolymers that may have arisen during sequencing by removing nucleotides that TMAP identified as insertions from the read. Haplotypes were then inferred based on the combination of nucleotide differences between the read and the reference sequence. The proportion of reads determined the prevalence of haplotypes within each individual as identified by their barcode sequence^29^. For this analysis, we defined variant positions empirically as those demonstrating polymorphism in the preceding allele variant analysis, in order to mitigate the risk of false-positive haplotype inference resulting from base-calling error in homopolymers. To avoid minority variants in highly sequenced parasitemias being over-represented, any individual whose number of reads was greater than three standard deviations from the mean was censored.

### Statistical analyses

In order to estimate the specificity of this new genotyping approach for variant allele and haplotype detection, we estimated false discovery rates (FDRs) from the Variant Calling Files (VCF) generated by the allele analysis. We computed these using reads derived from the control DNA mixtures separately for each gene target. FDRs were calculated from VCF files by summing the number of nucleotide positions containing a previously unidentified alternate allele while allowing for minor allele frequencies (MAFs) of 0.1%, 0.5%, 1%, 2%, 5%, and 10%. FDR results were expressed as the number of false positive SNPs per individual. We compared the expected and observed frequencies of alleles and haplotypes in the reads obtained from control DNA mixtures using Pearson product-moment correlation coefficients. The divergence from this perfect concordance was analyzed with 95% confidence intervals and two-tailed P tests demonstrated significance at p < 0.0001 for all comparisons. Multiplicity of infection (MOI) was defined as number of unique haplotypes of a single gene that was observed within an individual infection^30^. MOI values were compared between groups using either the Mann Whitney U test or Kruskal Wallis test.

### Ethics

The field study in Kenya was approved by the research ethics committees of Moi University and Duke University. All participants provided written informed consent prior to participation in the study. All experiments were performed in accordance with relevant guidelines and regulations.

## Results

### Yield of multiplex target sequencing of complex templates

**Figure 2:**
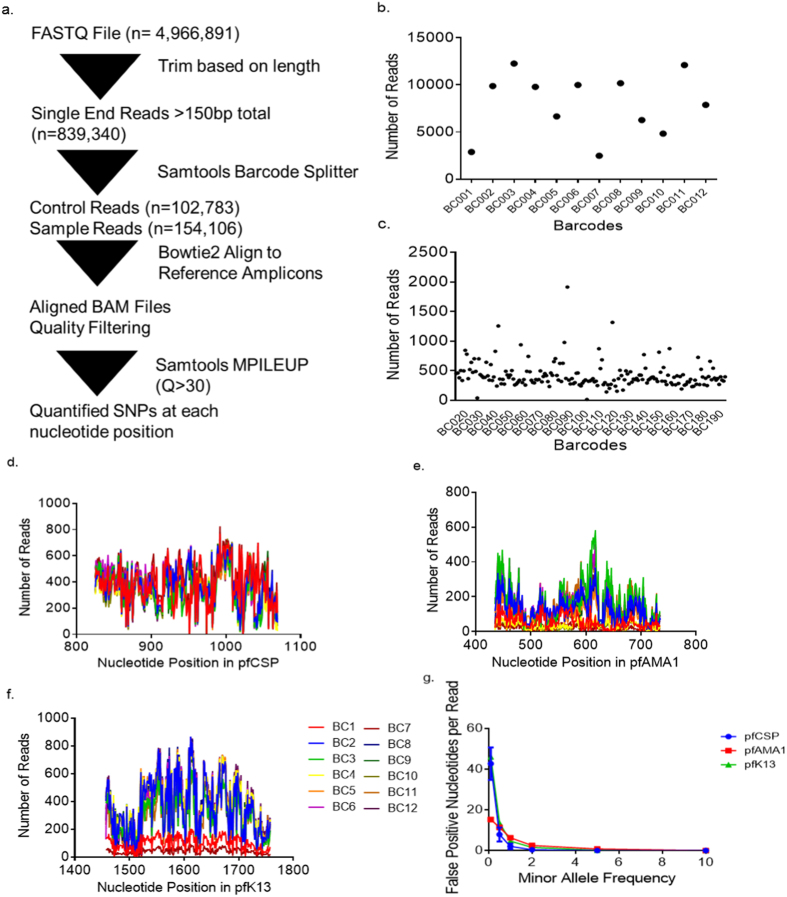
Performance of NGS Technology. (**a**) Bioinformatic pipeline for analysis of individual reads acquired from multiplex sequencing on an Ion Torrent 318 chip. (**b**) Number of reads assigned to each barcode in control DNA mixtures. (**c**) Number of reads assigned to each barcode in field-collected parasites. Coverage map across amplicon for *pf-csp* (**d**), *pf-ama1* (**e**), and *pf-k13* (**f**,**g**). False discovery rates at varying minor allele frequencies for reads from each gene target.

We sequenced PCR products in a single Ion Torrent reaction from three gene targets generated from 192 gDNA templates: six control DNA mixtures in duplicate and 180 field-collected DNA samples. The control DNA mixtures comprised gDNA of up to six *P. falciparum* reference lines in varying proportions (Table 1A). The combined sequencing run initially yielded 4,966,891 raw reads, of which 839,340 were ≥150 bp. 102,783 reads derived from control templates and 154,106 reads derived from field parasites; the balance of reads failed criteria for barcode matching. The median number of analyzable reads was 8,836 (range 2485-12,268) for control mixtures (Fig. 2b) and 363 (range 14–1,916) for field-collected DNA samples (Fig. 2c).

When mapped to reference sequences for *pf-csp, pf-ama1*, or *pf-k13* and considering only base calls with high quality, base coverage varied substantially both between and within amplicons. The median (IQR) coverage was 747 (724–784) for *pf-csp*, 344 (155–379) for *pf-ama1*, and 746 (729–753) for *pf-k13* (Fig. 2d,e and f), likely owing to indels that were prevalent around homopolymeric stretches.

### False discovery rate

In order to evaluate the specificity of the laboratory and analytic approach, we computed FDRs for each gene target by calculating the likelihood of recovering false allele variants at any given position across a range of MAFs (Fig. 2g). A very low MAF of 0.1% would have returned an average of 93 (*pf-csp)*, 36 (*pf-ama1)* or 85 (*pf-k13)* false alleles per amplicon. In contrast, at a MAF of 2% the FDR for each gene target was less than 1 nucleotide per amplicon (Fig. 2g). Therefore, consistent with prior deep amplicon sequencing studies of *P. falciparum*^31^, we enforced for subsequent analyses an MAF lower limit of 2%.

### Allele frequency estimation within complex templates

We first examined allele frequencies of *pf-csp, pf-ama1*, and *pf-k13* variants in reads derived from control DNA mixtures. From these field-collected DNA samples, the number of mapped sequences was 60,548 for *pf-csp*, 10,904 for *pf-ama1*, and 17,595 for *pf-k13*. As noted above, base coverage within amplicons varied reproducibly between libraries when low-quality base calls were censored, likely owing to reduced quality scores in homopolymeric segments of each amplicon (Fig. 2d,e and f). Overall, of 102,783 reads that were identified by barcode, 89,047 could ultimately be mapped.

The frequency of observed SNPs was calculated at each position in the *pf-csp, pf-ama1* and *pf-k13* amplicons for each of the control pools (Figure S2). We observed allelic variation at expected sites in *pf-csp* and *pf-ama1* consistent with known haplotypes in the reference parasite lines; in contrast, *pf-k13* demonstrated no variation in the control mixtures, owing to invariant K13 templates in the reference parasite lines.

**Figure 3:**
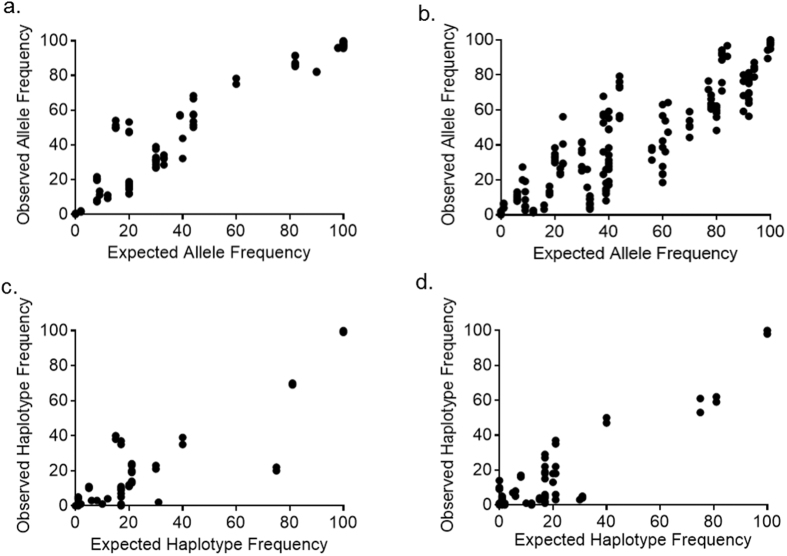
Performance of multiplex amplification and sequencing of control DNA mixtures. Expected compared to observed frequency of individual SNPs (alleles) in reads of *pf-csp* (**a**) and *pf-ama1* (**b**) P values are p < 0.0001 and r-values are r = 0.9532 and r = 0.8387 for *pf-csp* and *pf-ama1*. Expected vs observed frequency of haplotypes in reads of *pf-csp* (**c**) and *pf-ama1* (**d**) P values are p < 0.0001 and r-values are r = 0.7333 and r = 0.8085 for *pf-csp* and *pf-ama1*.

We compared expected and observed allele frequencies at each variant position in *pf-csp* (Fig. 3a) and *pf-ama1* (Fig. 3b). The correlations were high for each target between expected and observed allele frequencies for variant gene targets (each p < 0.0001 by Pearson’s PMCC), with r-values of 0.9532 (*pf-csp)* and 0.8387 (*pf-ama1)*. We did observe outliers in observed frequencies of alleles, which were not associated with specific mixtures, reference lines, or homopolymeric segments of the gene targets. Overall, therefore, this laboratory and analytic approach provided precise quantitation of variant alleles in multiple targets present in complex templates.

### Haplotype inference in control DNA mixtures

Variant positions identified in allele frequency analysis were then used to construct haplotypes of each gene target. Overall, the control samples produced a total of 90,766 reads from 3 gene targets (*pf-ama1:* 10,904, *pf-csp:* 62,650, *pf-k13:* 17,650); the median number of reads per control infection were 1,007 (*pf-ama1),* 5,234 (*pf-csp)* and 1,273 (*pf-k13*). Expected and observed haplotype frequencies were highly correlated for the two polyclonal gene targets (*pf-csp* and *pf-ama1* each p < 0.0001) with r-values of 0.7333 (*pf-csp*) and 0.8085 (*pf-ama1)* (Fig. 3c and d). These lower r-values (compared to those for the cognate allele-based analysis) indicate greater deviance from expected frequencies for haplotypes than for alleles, possibly owing to a lower number of observations input into the haplotype analysis.

### Yield and allelic diversity of multiplex target sequencing of field-collected parasite DNA

Amplicons from *pf-csp, pf-ama1*, and *pf-k13* that were generated from 180 field-collected parasite DNA samples were sequenced with the above products in a single Ion Torrent sequencing run. Each individual field-collected DNA sample generated a median of 363 reads (range 126–17,362) (Fig. 2d). We first identified variant alleles in the three gene targets in the field-collected DNA samples (as above) in order to generate an array of validated, quality-filtered variant positions. We observed no statistically significant variation in the sequenced portion of *pf-k13;* notably, we did not observe the Y493H, R539T, or C580Y substitutions that confer artemisinin-resistance in SE Asian parasites^31^,^32^,^33^. In contrast, using minimum base-call quality scores of 25 and an MAF of 2% to minimize false-discovery, we observed 25 variant positions in *pf-csp* and 30 variant positions in *pf-ama1* (Table S2).

### Haplotype inference in field-collected parasites

We used these validated variant positions in each gene target to assign gene-specific haplotypes using the same approach as that used for control DNA mixtures. For consideration in this analysis, a minimum of 10 reads was required to support each individual haplotype. Using this approach, we observed 35 *pf-csp* haplotypes within a subset of 45,817 reads and 13 *pf-ama1* unique haplotypes among 526 reads. Differences between targets in the abundance of analyzable reads for haplotypes resulted from fewer overall reads obtained for *pf-ama*1, possibly resulting from lower amplification efficiency.

**Figure 4:**
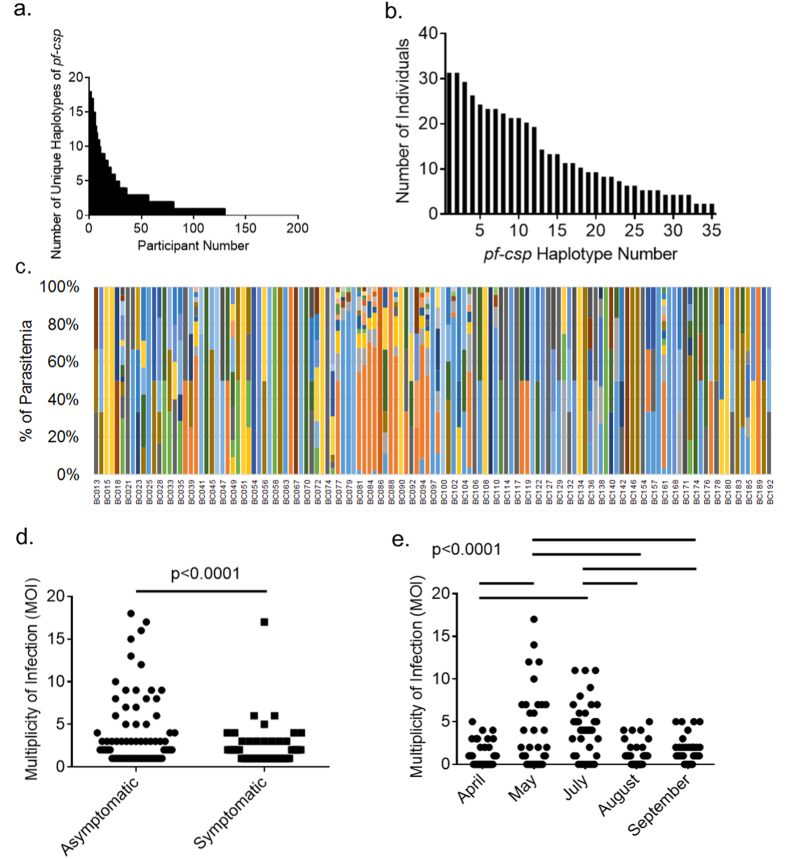
Haplotypes present in clinical infection samples. (**a**) Number of unique *pf-csp* haplotypes contained in field-collected sample DNA by individual, sorted by barcode number. (**b**) Number of individuals bearing each *pf-csp* haplotype. (**c**) Distribution of *pf-csp* haplotypes among field-collected sample DNA. Each color indicates a different haplotype. (**d**) MOI values segregated based upon symptomaticity. (**e**) MOI values segregated based upon sampling date.

We recovered high quality haplotypes in 135 individuals for *pf-csp* and 56 individuals for *pf-ama1*. Among these individuals, more than one unique haplotype was identified in 64% of the individuals (n = 86) at *pf-csp* and in 18% (n = 10) at *pf-ama1*; the median number of observed haplotypes per individual was 2 (range 1–18) for *pf-csp* and 1 (range 1–12) for *pf-ama1* while the remaining individuals contained haplotypes that were not supported by our quality criteria (Fig. 4a).

The number of individuals bearing each of the 35 *pf-csp* haplotypes were calculated for all shared haplotypes (Fig. 4b). The median number of patients sharing each haplotype was 10 (range 2–31). Of the 35 *pf-csp* haplotypes that we observed, 3 haplotypes were present in only 2 individuals, 4 haplotypes were present in only 4 individuals and 28 haplotypes were present in 5 or more individuals. There was no clear correlation between the number of *pf-csp* and *pf-ama1* haplotypes in the 43 individuals in whom we identified haplotypes at both loci possibly owing to the lower median MOI in these individuals when measured by *pf-ama1* (median = 1) than by *pf-csp* (median = 2) (Figure S4). The composition of these haplotypes were determined and the percent of reads supporting each haplotype for each individual was calculated (Fig. 4c). Using these *pf-csp* haplotypes as an index of MOI, we compared MOI values between clinical and demographic groups. There were no discernable differences in MOI between participants based on gender, age, or geographic location (Figure S3A–C). However, median MOI was significantly higher in asymptomatic (median = 3) than in symptomatic (median = 2) participants (Mann Whitney U test p < 0.0001 and Kruskal Wallis Test p < 0.0001). Similarly, we observed significant differences in median MOI across sampling dates, with the highest median MOI in May and July and the lowest in April, August and September (p < 0.0001), suggesting that this rapid approach to haplotype inference may be useful for tracking parasite populations as a surveillance tool over time.

## Discussion

NGS approaches to parasite genotyping allow for the sensitive detection of minority variants, disaggregation of complex parasite mixtures, and scalable processing of large sample sets. Therefore, we designed, validated, and applied an overlap extension barcoding and multiplexing NGS approach to parasite genotyping that leverages NGS of individually barcoded samples in a scalable, modular, and multiplex manner. In control mixtures of reference parasite genomes, we qualitatively and quantitatively detected unique haplotypes of more than one gene target comprising as little as 2% of polyclonal infections. We then applied our genotyping approach to field-collected samples collected in Western Kenya in order to rapidly obtain genotypes at three unlinked loci and reconstruct haplotypes of diverse markers. Our overlap extension NGS genotyping strategy therefore enables a rapid, flexible, high-throughput method for genotyping of individual *P. falciparum* parasitemias that can enhance molecular epidemiologic studies of parasite gene markers, population structure, and transmission.

Targeted amplicon sequencing is useful in examining a small number of gene targets in a large number of individuals, but requires the addition of identifying barcodes for each sample in order to disambiguate the resulting sequence reads. Two traditional approaches to barcoding amplicons are the amplification of gene targets using fusion primers that contain target-specific sequence as well as barcodes^34^ or the post-amplification enzymatic ligation of barcodes to amplicons^34^. The scalability of these approaches is undermined by the needs for additional labor and downstream purification, reduced amplification efficiency, or increased reagent costs. As an alternative, a scalable and modular approach was developed that allowed for the separate generation of barcodes and of gene target amplicons, followed by a secondary amplification reaction of limited cycle number to fuse these two products^35^,^36^; this approach has been applied previously to studies of microbiota^35^,^36^ molecular ecology^37^, and phylogenetics^38^. This “molecular barcoding” approaches have allowed for a reduction in false positives and enabled sensitive screening for single nucleotide variation compared to previous sample preparation methods^39^. This approach was successfully applied to malaria to identify *pf-csp* haplotypes in individuals treated with the RTS,S/AS01 vaccine^9^. Owing to our need to process large numbers of field parasites at multiple targets, we used a similar modular approach, and included multiple gene targets to avoid the amplicon homogeneity and generate genotype data relevant for multiple analyses.

The overlap extension barcoding and NGS approach precisely and sensitively quantified SNPs in multiple gene targets in complex mixtures of parasites. The sensitivity of this approach is evidenced by the low false discovery rate that identified fewer than one incorrectly called SNP per gene target when allowing for minor allele frequency of 2%. In addition, the precision of this approach is supported by the high concordance (p < 0.0001 and r = 0.9532 for *pf-csp*) between expected and observed frequencies of SNPs in the control DNA mixtures (Fig. 2a and b). This high analytical sensitivity and specificity is important because, in high transmission areas, most parasitemias comprise many unique parasite genomes^6^,^17^,^18^,^40^ and traditional genotyping approaches may either fail to generate analyzable data owing to complex templates or fail to capture minority variant subpopulations; these minority variant populations can harbor clinically-important genotypes^41^. The correlations between observed and expected allele frequencies in control DNA samples for *pf-ama1* were lower than those reported in studies employing whole genome sequencing of polygenomic templates^42^. This overrepresentation of some alleles may result from bias in either PCR amplification or successful sequencing of specific templates, or from generation of chimeras at a low frequency. Alternatively, PCR bias may have generated chimeras at a low frequency which could include segments of amplicons from different parasites which would appear as incorrect base determinations. The parallel processing of specimens and gene targets allows overlap extension barcoding to genotype and sequence parasites without the inconsistency and cost of traditional approaches^43^,^44^,^45^.

The sensitivity and specificity of this approach allowed for the rapid measurement in field-collected *P. falciparum* samples of SNPs that are directly relevant to treatment and prevention. Firstly, artemisinin resistance in Southeast Asian *P. falciparum* is conferred by SNPs in the *pf-k13* gene target, and surveillance for pathogenic mutations in parasite populations is underway^31^,^32^,^33^. In our study, the resistance-conferring Y493H, R539T, and C580Y substitutions were absent, which is consistent with prior studies^31^,^32^. Surprisingly, we did not observe the K13 variation that has been reported in other studies, despite the large diversity of *pf-csp* and *pf-ama1* alleles. Secondly, we genotyped parasites at *pf-csp*, which is targeted by the partially-effective RTS,S/AS01 monovalent *P. falciparum* vaccine: because *pf-csp* is very diverse and only the 3D7 haplotype is included in the vaccine, the greatest protection is conferred against parasites with matching (i.e. 3D7) haplotypes^9^. Of the 135 field-collected sample DNA from which we successfully inferred *pf-csp* haplotypes, only 13 participants (9.6%) harbored the 3D7 *pf-csp* haplotype; this prevalence is similar to that in other settings^9^, and indicates a high prevalence of potentially vaccine-resistant *P. falciparum* strains^9^. Future applications of this modular approach to parasite genotyping may include additional markers for drug- and vaccine-resistance to enable more efficient deployment of control measures and program assessment.

Overlap extension barcoding followed by NGS allowed for the inference of haplotypes for both artificial mixtures of parasite genomes and field-collected sample DNA. In the mixtures of control DNA, we observed high correlation between expected and observed frequencies of haplotypes for both *pf-csp* and *pf-ama1* (Fig. 3c and d). For field-collected sample DNA, *pf-csp* contained 35 haplotypes, composed of SNPs that were identified by allele analysis, and both semi-private (haplotypes limited to only 2 patients) and highly shared haplotypes were noted (Fig. 4c). Given 1) the ability to sensitively and precisely quantitate diverse haplotypes in polyclonal field-collected sample DNA, 2) the observed diversity of *pf-csp* in the field-collected sample DNA, and 3) the high degree of haplotype sharing between infected individuals, this approach can be useful to interrogate polymorphic markers as a means to characterize the population structure of *P. falciparum*. The rapid surveillance of *P. falciparum* population structure can enable efficient monitoring of malaria control program effectiveness, detection of imported genotypes, and parasite population expansion^46^.

In contrast to pooled deep sequencing approaches^31^,^47^ overlap extension barcoding generates sequencing data can be disaggregated and assigned to individual patients. While pooled deep sequencing approaches allow ecological studies of the genetic epidemiology of parasites^31^,^47^, they cannot link parasite genotypes with clinical phenotypes of drug response, virulence, or transmission. Overlap extension barcoding is a logical way to broaden the utility of NGS to allow for individual-level comparisons between individuals within the population based upon clinical and demographic criteria (Fig. 2c). Given clinical and demographic information (Table S3), we were able to separate the patients into biologically meaningful groups such as location, age, gender, sampling date and symptomaticity (Figure S3). Using haplotype inference at *pf-csp* as an index of MOI, we observed higher MOI values in asymptomatic individuals and variation in MOI values based on sampling date. The increase in MOI is consistent with the observation that rainfall is highly correlated with malarial disease burden in this region and the months associated with the highest MOI values correlate with the heaviest rainfall in this area^48^,^49^. Thus by disaggregating patient data into biologically meaningful groups, we might be able to draw conclusions about between these factors and transmission patterns of malaria.

Our approach has potential limitations. As with any PCR protocol involving post-amplification processing steps, there is potential for cross-contamination. Therefore, we employed the requisite strict separation of processive steps and inclusion of appropriate positive and negative controls in each step. Additionally, we obtained variable read yield between field-collected sample DNA owing to differing quantities of *P. falciparum* gDNA. This could be mitigated by estimating amplicon concentration and altering template volumes on some samples prior to the generation of sequencing libraries. The *pf-ama1* amplification produced fewer reads than either *pf-csp* or *pf-k13*; efficiency may be increased by using primers with higher melting temperature or less internal base pairing. Efficiency in this approach was sub-optimal due largely to reads that were censored for length, a phenomenon also noted by other groups using a similar method^37^ which might be mitigated during library preparation by more stringent size-selection and purification prior to sequencing. In order to offset this observed inefficiency and obtain a large number of analyzable reads, we employed higher yield sequencing chips on the Ion Torrent platform. Finally, in common with other NGS protocols, the generation of chimeric sequences complicates assessments of genetic diversity^50^. In order to mitigate this problem, we gel-purified the full length sequencing product and used particularly high melting temperatures during the overlap extension PCR step to avoid spurious priming.

Overlap extension barcoding may prove useful in a number of different applications. The highly modular nature of the approach allows it to be easily adapted for alternate gene targets: Constellations of gene targets that mediate drug resistance, vaccine resistance, or virulence can be catalogued scalably from individual infections. Alternatively, neutral markers can be surveilled in order to detect changes in parasite population structure in response to control measures, or to construct fine-scale transmission networks of *P. falciparum* in order to understand human-mosquito transmission. The efficient, rapid, and modular nature of this NGS approach to parasite genotyping may enable broader adoption of deep sequencing to field studies of parasite epidemiology.

## Additional Information

**How to cite this article**: Levitt, B. *et al*. Overlap Extension Barcoding for the Next Generation Sequencing and Genotyping of Plasmodium falciparum in Individual Patients in Western Kenya. *Sci. Rep.* **7**, 41108; doi: 10.1038/srep41108 (2017).

**Publisher's note:** Springer Nature remains neutral with regard to jurisdictional claims in published maps and institutional affiliations.

## Supplementary Material

Supplementary Information

## Figures and Tables

**Table 1:** (a) Composition of control pools 1–6 from lab strains 3D7, DD2, HB3, 7G8, K1 and FCR3, (b) SNP identities for various positions of *pf-csp* in each lab strain of *Plasmodium falciparum*.

	3D7	DD2	HB3	7G8	K1	FCR3
(**a**)						
Control1	100%	0%	0%	0%	0%	0%
Control2	17%	17%	17%	17%	17%	17%
Control3	1%	6%	81%	12%	0%	0%
Control4	20%	80%	8%	2%	15%	75%
Control5	31%	21%	21%	21%	5%	1%
Control6	10%	20%	40%	30%	0%	0%
(**b**)						
G862A	G	G	G	A	G	G
G902A	G	A	A	A	A	A
C905A	C	C	C	C	A	C
A949G	A	G	G	G	G	G
G952C	G	C	C	C	C	C
C963G	C	G	G	G	G	G
C970A	C	C	A	C	C	C
A973T	A	A	A	A	T	A
C979A	C	C	A	C	C	C
A1054G	A	A	A	G	G	G
A1055G	A	A	A	G	A	A
C1060T	C	C	C	T	C	C
