# Supplementary material for: Overlap Extension Barcoding for the Next Generation Sequencing and Genotyping of Plasmodium falciparum in Individual Patients in Western Kenya
Source: Sci Rep. 2017 Jan 24;7:41108. doi: 10.1038/srep41108 (PMC5259759; doi:10.1038/srep41108)
Supplement: Supplementary Information [file srep41108-s1.pdf]

Overlap Extension Barcoding for the Next Generation Sequencing and Genotyping of Plasmodium Falciparum in Individual Patients in Western Kenya

Brandt Levitt B.S. [1], Andrew Obala Ph.D. [2], Scott Langdon Ph.D. [3], David Corcoran Ph.D. [4], Wendy Prudhomme O'Meara Ph.D. [5], Steve M Taylor M.D. M.P.H* [5]

1 Department of Molecular Genetics and Microbiology, Duke University Medical Center, Durham, NC, USA

2 Moi University School of Medicine, College of Health Sciences, Nandi Rd, Eldoret, Kenya

3 Duke University DNA Analysis Facility, Department of Immunology, Duke University Medical Center, Durham, NC, USA

4 Duke Center for Genomic and Computational Biology, Duke University Medical Center, Durham NC, USA

5 Division of Infectious Diseases and Duke Global Health Institute, Duke University Medical Center, Durham, NC, USA

*Corresponding Author. Correspondence to Steve.Taylor@duke.edu

Primer Name	Sequence (5'-3')	Barcode
IonTorrent Adaptor A forward	CCATCTCATCCCTGCGTGTCTCCGACTCAG	
Reverse Complement Linker Sequence	TCACCGCTCGGATC	
Reverse Complement Adaptor Sequence	CTGAGTCGGAGACACGCA	
IonTorrent Adaptor A reverse	TCACCGCTCGGATC/XXXXXXXXXX/CTGAGTCGGAGACACGCA	
IonTorrent Adaptor A reverse Barcode 001	TCACCGCTCGGATC/GTTACCTTAG/CTGAGTCGGAGACACGCA	CTAAGGTAAC
IonTorrent Adaptor A reverse Barcode_002	TCACCGCTCGGATC/GTTCTCCTTA/CTGAGTCGGAGACACGCA	TAAGGAGAAC
IonTorrent Adaptor A reverse Barcode 003	TCACCGCTCGGATC/GAATCCTCTT/CTGAGTCGGAGACACGCA	AAGAGGATTC
IonTorrent Adaptor A reverse Barcode 004	TCACCGCTCGGATC/GATCTTGGTA/CTGAGTCGGAGACACGCA	TACCAAGATC
IonTorrent Adaptor A reverse Barcode 005	TCACCGCTCGGATC/GTTCCTTCTG/CTGAGTCGGAGACACGCA	CAGAAGGAAC
IonTorrent Adaptor A reverse Barcode 006	TCACCGCTCGGATC/GAACTTGCAG/CTGAGTCGGAGACACGCA	CTGCAAGTTC
IonTorrent Adaptor A reverse Barcode_007	TCACCGCTCGGATC/GAATCACGAA/CTGAGTCGGAGACACGCA	TTCGTGATTC
IonTorrent Adaptor A reverse Barcode 008	TCACCGCTCGGATC/GTTATCGGAA/CTGAGTCGGAGACACGCA	TTCCGATAAC
IonTorrent Adaptor A reverse Barcode 009	TCACCGCTCGGATC/GTTCCGCTCA/CTGAGTCGGAGACACGCA	TGAGCGGAAC
IonTorrent Adaptor A reverse Barcode 010	TCACCGCTCGGATC/GTTCGGTCAG/CTGAGTCGGAGACACGCA	CTGACCGAAC
IonTorrent Adaptor A reverse Barcode 011	TCACCGCTCGGATC/GATTCGAGGA/CTGAGTCGGAGACACGCA	TCCTCGAATC
IonTorrent Adaptor A reverse Barcode 012	TCACCGCTCGGATC/GAACCACCTA/CTGAGTCGGAGACACGCA	TAGGTGGTTC
IonTorrent Adaptor A reverse Barcode 013	TCACCGCTCGGATC/GTCCGTTAGA/CTGAGTCGGAGACACGCA	TCTAACGGAC
IonTorrent Adaptor A reverse Barcode 014	TCACCGCTCGGATC/GACACTCCAA/CTGAGTCGGAGACACGCA	TTGGAGTGTC
IonTorrent Adaptor A reverse Barcode 015	TCACCGCTCGGATC/GACCTCTAGA/CTGAGTCGGAGACACGCA	TCTAGAGGTC
IonTorrent Adaptor A reverse Barcode 016	TCACCGCTCGGATC/GTCATCCAGA/CTGAGTCGGAGACACGCA	TCTGGATGAC
IonTorrent Adaptor A reverse Barcode 017	TCACCGCTCGGATC/GACGAATAGA/CTGAGTCGGAGACACGCA	TCTATTCGTC
IonTorrent Adaptor A reverse Barcode 018	TCACCGCTCGGATC/GCAATTGCCT/CTGAGTCGGAGACACGCA	AGGCAATTGC
IonTorrent Adaptor A reverse Barcode_019	TCACCGCTCGGATC/GTCCGACTAA/CTGAGTCGGAGACACGCA	TTAGTCGGAC
IonTorrent Adaptor A reverse Barcode 020	TCACCGCTCGGATC/GATGGATCTG/CTGAGTCGGAGACACGCA	CAGATCCATC
IonTorrent Adaptor A reverse Barcode 021	TCACCGCTCGGATC/GTAATTGCGA/CTGAGTCGGAGACACGCA	TCGCAATTAC
IonTorrent Adaptor A reverse Barcode 022	TCACCGCTCGGATC/GCGTCTCGAA/CTGAGTCGGAGACACGCA	TTCGAGACGC
IonTorrent Adaptor A reverse Barcode 023	TCACCGCTCGGATC/GTTCGTGGCA/CTGAGTCGGAGACACGCA	TGCCACGAAC
IonTorrent Adaptor A reverse Barcode_024	TCACCGCTCGGATC/GAATGAGGTT/CTGAGTCGGAGACACGCA	AACCTCATTC
IonTorrent Adaptor A reverse Barcode 025	TCACCGCTCGGATC/GTATCTCAGG/CTGAGTCGGAGACACGCA	CCTGAGATAC
IonTorrent Adaptor A reverse Barcode 026	TCACCGCTCGGATC/GAGGTTGTAA/CTGAGTCGGAGACACGCA	TTACAACCTC
IonTorrent Adaptor A reverse Barcode 027	TCACCGCTCGGATC/GCGGATGGTT/CTGAGTCGGAGACACGCA	AACCATCCGC
IonTorrent Adaptor A reverse Barcode 028	TCACCGCTCGGATC/GATTCCGGAT/CTGAGTCGGAGACACGCA	ATCCGGAATC
IonTorrent Adaptor A reverse Barcode 029	TCACCGCTCGGATC/GATAACCTCG/CTGAGTCGGAGACACGCA	CGAGGTTATC
IonTorrent Adaptor A reverse Barcode 030	TCACCGCTCGGATC/GCAGCTTGGA/CTGAGTCGGAGACACGCA	TCCAAGCTGC
IonTorrent Adaptor A reverse Barcode_031	TCACCGCTCGGATC/GTGTGTAAGA/CTGAGTCGGAGACACGCA	TCTTACACAC
IonTorrent Adaptor A reverse Barcode 032	TCACCGCTCGGATC/GTTCAATGAGAA/CTGAGTCGGAGACACGCA	TTCTCATTGAAC
IonTorrent Adaptor A reverse Barcode 033	TCACCGCTCGGATC/GAACGATGCGA/CTGAGTCGGAGACACGCA	TCGCATCGTTC
IonTorrent Adaptor A reverse Barcode 034	TCACCGCTCGGATC/GACAATGGCTTA/CTGAGTCGGAGACACGCA	TAAGCCATTGTC
IonTorrent Adaptor A reverse Barcode 035	TCACCGCTCGGATC/GACGATTCCTT/CTGAGTCGGAGACACGCA	AAGGAATCGTC
IonTorrent Adaptor A reverse Barcode 036	TCACCGCTCGGATC/GACATTCTCAAG/CTGAGTCGGAGACACGCA	CTTGAGAATGTC
IonTorrent Adaptor A reverse Barcode 037	TCACCGCTCGGATC/GTCCGTCCTCCA/CTGAGTCGGAGACACGCA	TGGAGGACGGAC
IonTorrent Adaptor A reverse Barcode 038	TCACCGCTCGGATC/GCCGATTGTTA/CTGAGTCGGAGACACGCA	TAACAATCGGC
IonTorrent Adaptor A reverse Barcode 039	TCACCGCTCGGATC/GATTATGTCAG/CTGAGTCGGAGACACGCA	CTGACATAATC
IonTorrent Adaptor A reverse Barcode 040	TCACCGCTCGGATC/GCGAAGTGGAA/CTGAGTCGGAGACACGCA	TTCCACTTCGC
IonTorrent Adaptor A reverse Barcode_041	TCACCGCTCGGATC/GATTCGTGCT/CTGAGTCGGAGACACGCA	AGCACGAATC
IonTorrent Adaptor A reverse Barcode 042	TCACCGCTCGGATC/GCTGGCCTCCAA/CTGAGTCGGAGACACGCA	TTGGAGGCCAGC
IonTorrent Adaptor A reverse Barcode 043	TCACCGCTCGGATC/GAGGAAGCTCCA/CTGAGTCGGAGACACGCA	TGGAGCTTCCTC
IonTorrent Adaptor A reverse Barcode 044	TCACCGCTCGGATC/GTTCGGACTGA/CTGAGTCGGAGACACGCA	TCAGTCCGAAC
IonTorrent Adaptor A reverse Barcode_045	TCACCGCTCGGATC/GTGGTTGCCTTA/CTGAGTCGGAGACACGCA	TAAGGCAACCAC
IonTorrent Adaptor A reverse Barcode 046	TCACCGCTCGGATC/GTCTCTTAGAA/CTGAGTCGGAGACACGCA	TTCTAAGAGAC
IonTorrent Adaptor A reverse Barcode 047	TCACCGCTCGGATC/GTTATGTTAGGA/CTGAGTCGGAGACACGCA	TCCTAACATAAC
IonTorrent Adaptor A reverse Barcode 048	TCACCGCTCGGATC/GCCATTGTCCG/CTGAGTCGGAGACACGCA	CGGACAATGGC
IonTorrent Adaptor A reverse Barcode 049	TCACCGCTCGGATC/GAATAGGCTCAA/CTGAGTCGGAGACACGCA	TTGAGCCTATTC
IonTorrent Adaptor A reverse Barcode 050	TCACCGCTCGGATC/GTTCCATGCGG/CTGAGTCGGAGACACGCA	CCGCATGGAAC
IonTorrent Adaptor A reverse Barcode 051	TCACCGCTCGGATC/GAGGATTGCCAG/CTGAGTCGGAGACACGCA	CTGGCAATCCTC
IonTorrent Adaptor A reverse Barcode 052	TCACCGCTCGGATC/GAGGAGGTGGA/CTGAGTCGGAGACACGCA	TCCACCTCCTC
IonTorrent Adaptor A reverse Barcode 053	TCACCGCTCGGATC/GAATTAATGCTG/CTGAGTCGGAGACACGCA	CAGCATTAATTC
IonTorrent Adaptor A reverse Barcode 054	TCACCGCTCGGATC/GAACATCAAGGA/CTGAGTCGGAGACACGCA	TCCTTGATGTTC
IonTorrent Adaptor A reverse Barcode_055	TCACCGCTCGGATC/GAAGAGCTAGA/CTGAGTCGGAGACACGCA	TCTAGCTCTTC
IonTorrent Adaptor A reverse Barcode 056	TCACCGCTCGGATC/GATCCGAGTGA/CTGAGTCGGAGACACGCA	TCACTCGGATC
IonTorrent Adaptor A reverse Barcode 057	TCACCGCTCGGATC/GTGAAGCAGGAA/CTGAGTCGGAGACACGCA	TTCCTGCTTCAC
IonTorrent Adaptor A reverse Barcode 058	TCACCGCTCGGATC/GAACTCTAAGG/CTGAGTCGGAGACACGCA	CCTTAGAGTTC
IonTorrent Adaptor A reverse Barcode 059	TCACCGCTCGGATC/GTCGGAACTCAG/CTGAGTCGGAGACACGCA	CTGAGTTCCGAC
IonTorrent Adaptor A reverse Barcode 060	TCACCGCTCGGATC/GATGTGCCAGGA/CTGAGTCGGAGACACGCA	TCCTGGCACATC
IonTorrent Adaptor A reverse Barcode 061	TCACCGCTCGGATC/GATGATTGCGG/CTGAGTCGGAGACACGCA	CCGCAATCATC
IonTorrent Adaptor A reverse Barcode_062	TCACCGCTCGGATC/GATAATGTTGG/CTGAGTCGGAGACACGCA	CCAACATTATC
IonTorrent Adaptor A reverse Barcode 063	TCACCGCTCGGATC/GAACTTCTTGA/CTGAGTCGGAGACACGCA	TCAAGAAGTTC
IonTorrent Adaptor A reverse Barcode 064	TCACCGCTCGGATC/GCCAATTGAA/CTGAGTCGGAGACACGCA	TTCAATTGGC
IonTorrent Adaptor A reverse Barcode 065	TCACCGCTCGGATC/GACCAGTAGG/CTGAGTCGGAGACACGCA	CCTACTGGTC

IonTorrent Adaptor A reverse Barcode_066	TCACCGCTCGGATC/GTCGGAGCCTCA/CTGAGTCGGAGACACGCA	TGAGGCTCCGAC
IonTorrent Adaptor A reverse Barcode 067	TCACCGCTCGGATC/GTGTGGCCTTCG/CTGAGTCGGAGACACGCA	CGAAGGCCACAC
IonTorrent Adaptor A reverse Barcode 068	TCACCGCTCGGATC/GACAGGCAGA/CTGAGTCGGAGACACGCA	TCTGCCTGTC
IonTorrent Adaptor A reverse Barcode 069	TCACCGCTCGGATC/GAACCGATCG/CTGAGTCGGAGACACGCA	CGATCGGTTC
IonTorrent Adaptor A reverse Barcode 070	TCACCGCTCGGATC/GTATTCCTGA/CTGAGTCGGAGACACGCA	TCAGGAATAC
IonTorrent Adaptor A reverse Barcode 071	TCACCGCTCGGATC/GAGGTTCTTCCG/CTGAGTCGGAGACACGCA	CGGAAGAACCTC
IonTorrent Adaptor A reverse Barcode_072	TCACCGCTCGGATC/GAATCGCTTCG/CTGAGTCGGAGACACGCA	CGAAGCGATTC
IonTorrent Adaptor A reverse Barcode 073	TCACCGCTCGGATC/GAGAATTGGCTG/CTGAGTCGGAGACACGCA	CAGCCAATTCTC
IonTorrent Adaptor A reverse Barcode 074	TCACCGCTCGGATC/GCCTGCCTTCGA/CTGAGTCGGAGACACGCA	TCGAAGGCAGGC
IonTorrent Adaptor A reverse Barcode 075	TCACCGCTCGGATC/GCGAATGGCAGG/CTGAGTCGGAGACACGCA	CCTGCCATTCGC
IonTorrent Adaptor A reverse Barcode 076	TCACCGCTCGGATC/GAATGTCCTAG/CTGAGTCGGAGACACGCA	CTAGGACATTC
IonTorrent Adaptor A reverse Barcode 077	TCACCGCTCGGATC/GTTATGGAAG/CTGAGTCGGAGACACGCA	CTTCCATAAC
IonTorrent Adaptor A reverse Barcode 078	TCACCGCTCGGATC/GTTGAGGCTGG/CTGAGTCGGAGACACGCA	CCAGCCTCAAC
IonTorrent Adaptor A reverse Barcode 079	TCACCGCTCGGATC/GAATAACCAAG/CTGAGTCGGAGACACGCA	CTTGGTTATTC
IonTorrent Adaptor A reverse Barcode 080	TCACCGCTCGGATC/GTCCAGCCAA/CTGAGTCGGAGACACGCA	TTGGCTGGAC
IonTorrent Adaptor A reverse Barcode 081	TCACCGCTCGGATC/GAAGTGTTCGG/CTGAGTCGGAGACACGCA	CCGAACACTTC
IonTorrent Adaptor A reverse Barcode 082	TCACCGCTCGGATC/GAGATTCAGGA/CTGAGTCGGAGACACGCA	TCCTGAATCTC
IonTorrent Adaptor A reverse Barcode_083	TCACCGCTCGGATC/GCCGTGGTTAG/CTGAGTCGGAGACACGCA	CTAACCACGGC
IonTorrent Adaptor A reverse Barcode 084	TCACCGCTCGGATC/GCATCCTTCCG/CTGAGTCGGAGACACGCA	CGGAAGGATGC
IonTorrent Adaptor A reverse Barcode 085	TCACCGCTCGGATC/GATTGGACAAG/CTGAGTCGGAGACACGCA	CTTGTCCAATC
IonTorrent Adaptor A reverse Barcode 086	TCACCGCTCGGATC/GCTTGTCGGA/CTGAGTCGGAGACACGCA	TCCGACAAGC
IonTorrent Adaptor A reverse Barcode_087	TCACCGCTCGGATC/GATCTGTCCG/CTGAGTCGGAGACACGCA	CGGACAGATC
IonTorrent Adaptor A reverse Barcode 088	TCACCGCTCGGATC/GCCGCCTCAAGG/CTGAGTCGGAGACACGCA	CCTTGAGGCGGC
IonTorrent Adaptor A reverse Barcode 089	TCACCGCTCGGATC/GAAGAGGAAGAA/CTGAGTCGGAGACACGCA	TTCTTCCTCTTC
IonTorrent Adaptor A reverse Barcode 090	TCACCGCTCGGATC/GATCTTGAAGAA/CTGAGTCGGAGACACGCA	TTCTTCAAGATC
IonTorrent Adaptor A reverse Barcode 091	TCACCGCTCGGATC/GACAGTTCCAAG/CTGAGTCGGAGACACGCA	CTTGGAACTGTC
IonTorrent Adaptor A reverse Barcode 092	TCACCGCTCGGATC/GATTCCGGCCGA/CTGAGTCGGAGACACGCA	TCGGCCGGAATC
IonTorrent Adaptor A reverse Barcode 093	TCACCGCTCGGATC/GAATTATCTCCA/CTGAGTCGGAGACACGCA	TGGAGATAATTC
IonTorrent Adaptor A reverse Barcode 094	TCACCGCTCGGATC/GTCCGGAATTCA/CTGAGTCGGAGACACGCA	TGAATTCCGGAC
IonTorrent Adaptor A reverse Barcode 095	TCACCGCTCGGATC/GACGGTGGCAAG/CTGAGTCGGAGACACGCA	CTTGCCACCGTC
IonTorrent Adaptor A reverse Barcode_096	TCACCGCTCGGATC/GTGAATTGTTAG/CTGAGTCGGAGACACGCA	CTAACAATTCAC
IonTorrent Adaptor A reverse Barcode 097	TCACCGCTCGGATC/GTTCATTGCGAA/CTGAGTCGGAGACACGCA	TTCGCAATGAAC
IonTorrent Adaptor A reverse Barcode 098	TCACCGCTCGGATC/GCCGTGCGGAA/CTGAGTCGGAGACACGCA	TTCCGCACGGC
IonTorrent Adaptor A reverse Barcode 099	TCACCGCTCGGATC/GCAATTGGCCAA/CTGAGTCGGAGACACGCA	TTGGCCAATTGC
IonTorrent Adaptor A reverse Barcode 100	TCACCGCTCGGATC/GTTGAACTAGA/CTGAGTCGGAGACACGCA	TCTAGTTCAAC
IonTorrent Adaptor A reverse Barcode 101	TCACCGCTCGGATC/GAATTCTTCTCA/CTGAGTCGGAGACACGCA	TGAGAAGAATTC
IonTorrent Adaptor A reverse Barcode 102	TCACCGCTCGGATC/GATGGTTGAGG/CTGAGTCGGAGACACGCA	CCTCAACCATC
IonTorrent Adaptor A reverse Barcode 103	TCACCGCTCGGATC/GAATCCAGCAGG/CTGAGTCGGAGACACGCA	CCTGCTGGATTC
IonTorrent Adaptor A reverse Barcode_104	TCACCGCTCGGATC/GAATTCCTGCCA/CTGAGTCGGAGACACGCA	TGGCAGGAATTC
IonTorrent Adaptor A reverse Barcode 105	TCACCGCTCGGATC/GAATCGAAGCG/CTGAGTCGGAGACACGCA	CGCTTCGATTC
IonTorrent Adaptor A reverse Barcode 106	TCACCGCTCGGATC/GCAATCTGGAA/CTGAGTCGGAGACACGCA	TTCCAGATTGC
IonTorrent Adaptor A reverse Barcode 107	TCACCGCTCGGATC/GAAGACTCCGGA/CTGAGTCGGAGACACGCA	TCCGGAGTCTTC
IonTorrent Adaptor A reverse Barcode 108	TCACCGCTCGGATC/GATGGATGTA/CTGAGTCGGAGACACGCA	TACATCCATC
IonTorrent Adaptor A reverse Barcode_109	TCACCGCTCGGATC/GTCGTGTTGC/CTGAGTCGGAGACACGCA	GCAACACGAC
IonTorrent Adaptor A reverse Barcode 110	TCACCGCTCGGATC/GAGAATTGCTTA/CTGAGTCGGAGACACGCA	TAAGCAATTCTC
IonTorrent Adaptor A reverse Barcode 111	TCACCGCTCGGATC/GAATGGATCAG/CTGAGTCGGAGACACGCA	CTGATCCATTC
IonTorrent Adaptor A reverse Barcode 112	TCACCGCTCGGATC/GATTGTTCCTA/CTGAGTCGGAGACACGCA	TAGGAACAATC
IonTorrent Adaptor A reverse Barcode 113	TCACCGCTCGGATC/GAATTCCGGTT/CTGAGTCGGAGACACGCA	AACCGGAATTC
IonTorrent Adaptor A reverse Barcode 114	TCACCGCTCGGATC/GATTACCTCCGG/CTGAGTCGGAGACACGCA	CCGGAGGTAATC
IonTorrent Adaptor A reverse Barcode 115	TCACCGCTCGGATC/GAAGGTCCTGAA/CTGAGTCGGAGACACGCA	TTCAGGACCTTC
IonTorrent Adaptor A reverse Barcode 116	TCACCGCTCGGATC/GCATTGGTTAGA/CTGAGTCGGAGACACGCA	TCTAACCAATGC
IonTorrent Adaptor A reverse Barcode 117	TCACCGCTCGGATC/GATCAGCTCGGA/CTGAGTCGGAGACACGCA	TCCGAGCTGATC
IonTorrent Adaptor A reverse Barcode 118	TCACCGCTCGGATC/GAACATGGTAA/CTGAGTCGGAGACACGCA	TTACCATGTTC
IonTorrent Adaptor A reverse Barcode 119	TCACCGCTCGGATC/GACCGGAATGAG/CTGAGTCGGAGACACGCA	CTCATTCCGGTC
IonTorrent Adaptor A reverse Barcode_120	TCACCGCTCGGATC/GCCAGGCCTCGA/CTGAGTCGGAGACACGCA	TCGAGGCCTGGC
IonTorrent Adaptor A reverse Barcode 121	TCACCGCTCGGATC/GCAACCTTCCA/CTGAGTCGGAGACACGCA	TGGAAGGTTGC
IonTorrent Adaptor A reverse Barcode 122	TCACCGCTCGGATC/GTCGGAATCCTA/CTGAGTCGGAGACACGCA	TAGGATTCCGAC
IonTorrent Adaptor A reverse Barcode 123	TCACCGCTCGGATC/GCGGAGCTTCAA/CTGAGTCGGAGACACGCA	TTGAAGCTCCGC
IonTorrent Adaptor A reverse Barcode 124	TCACCGCTCGGATC/GAAGAAGTTGAA/CTGAGTCGGAGACACGCA	TTCAACTTCTTC
IonTorrent Adaptor A reverse Barcode 125	TCACCGCTCGGATC/GTTGAGCCTAA/CTGAGTCGGAGACACGCA	TTAGGCTCAAC
IonTorrent Adaptor A reverse Barcode_126	TCACCGCTCGGATC/GATTCGCCTTGG/CTGAGTCGGAGACACGCA	CCAAGGCGAATC
IonTorrent Adaptor A reverse Barcode 127	TCACCGCTCGGATC/GACCGATCTAAG/CTGAGTCGGAGACACGCA	CTTAGATCGGTC
IonTorrent Adaptor A reverse Barcode 128	TCACCGCTCGGATC/GAATCGGACCGG/CTGAGTCGGAGACACGCA	CCGGTCCGATTC
IonTorrent Adaptor A reverse Barcode 129	TCACCGCTCGGATC/GTCGCTCCAA/CTGAGTCGGAGACACGCA	TTGGAGCGAC
IonTorrent Adaptor A reverse Barcode 130	TCACCGCTCGGATC/GCCGGAACAAG/CTGAGTCGGAGACACGCA	CTTGTTCCGGC
IonTorrent Adaptor A reverse Barcode 131	TCACCGCTCGGATC/GATCTTGCCGGA/CTGAGTCGGAGACACGCA	TCCGGCAAGATC
IonTorrent Adaptor A reverse Barcode_132	TCACCGCTCGGATC/GTCGGATAGGAA/CTGAGTCGGAGACACGCA	TTCCTATCCGAC
IonTorrent Adaptor A reverse Barcode 133	TCACCGCTCGGATC/GATTCAATTAG/CTGAGTCGGAGACACGCA	CTAATTGAATC
IonTorrent Adaptor A reverse Barcode 134	TCACCGCTCGGATC/GTGGTGTCGAA/CTGAGTCGGAGACACGCA	TTCGACACCAC
IonTorrent Adaptor A reverse Barcode 135	TCACCGCTCGGATC/GCATGGCGGA/CTGAGTCGGAGACACGCA	TCCGCCATGC
IonTorrent Adaptor A reverse Barcode 136	TCACCGCTCGGATC/GAGGAACTGG/CTGAGTCGGAGACACGCA	CCAGTTCCTC
IonTorrent Adaptor A reverse Barcode 137	TCACCGCTCGGATC/GAATTATTGTTA/CTGAGTCGGAGACACGCA	TAACAATAATTC
IonTorrent Adaptor A reverse Barcode 138	TCACCGCTCGGATC/GATCCAGGCA/CTGAGTCGGAGACACGCA	TGCCTGGATC
IonTorrent Adaptor A reverse Barcode 139	TCACCGCTCGGATC/GTCCGACTTCAG/CTGAGTCGGAGACACGCA	CTGAAGTCGGAC

IonTorrent Adaptor A reverse Barcode_140	TCACCGCTCGGATC/GTTCCATTCCTT/CTGAGTCGGAGACACGCA	AAGGAATGGAAC
IonTorrent Adaptor A reverse Barcode 141	TCACCGCTCGGATC/GTCGGTTCGGAA/CTGAGTCGGAGACACGCA	TTCCGAACCGAC
IonTorrent Adaptor A reverse Barcode 142	TCACCGCTCGGATC/GATCCTGGTGAA/CTGAGTCGGAGACACGCA	TTCACCAGGATC
IonTorrent Adaptor A reverse Barcode 143	TCACCGCTCGGATC/GAAGTTGTAG/CTGAGTCGGAGACACGCA	CTACAACTTC
IonTorrent Adaptor A reverse Barcode 144	TCACCGCTCGGATC/GTGATGCCTCAG/CTGAGTCGGAGACACGCA	CTGAGGCATCAC
IonTorrent Adaptor A reverse Barcode 145	TCACCGCTCGGATC/GAATGATGCTGG/CTGAGTCGGAGACACGCA	CCAGCATCATTC
IonTorrent Adaptor A reverse Barcode_146	TCACCGCTCGGATC/GTTCAAGCCGG/CTGAGTCGGAGACACGCA	CCGGCTTGAAC
IonTorrent Adaptor A reverse Barcode 147	TCACCGCTCGGATC/GAATCTGCCTGA/CTGAGTCGGAGACACGCA	TCAGGCAGATTC
IonTorrent Adaptor A reverse Barcode 148	TCACCGCTCGGATC/GATCGTGCAGAA/CTGAGTCGGAGACACGCA	TTCTGCACGATC
IonTorrent Adaptor A reverse Barcode 149	TCACCGCTCGGATC/GTTATCTTCGGA/CTGAGTCGGAGACACGCA	TCCGAAGATAAC
IonTorrent Adaptor A reverse Barcode 150	TCACCGCTCGGATC/GAACGATGAGG/CTGAGTCGGAGACACGCA	CCTCATCGTTC
IonTorrent Adaptor A reverse Barcode 151	TCACCGCTCGGATC/GTTGGTTGCA/CTGAGTCGGAGACACGCA	TGCAACCAAC
IonTorrent Adaptor A reverse Barcode 152	TCACCGCTCGGATC/GACCGGATTCCG/CTGAGTCGGAGACACGCA	CGGAATCCGGTC
IonTorrent Adaptor A reverse Barcode 153	TCACCGCTCGGATC/GCTTCCTCAAGA/CTGAGTCGGAGACACGCA	TCTTGAGGAAGC
IonTorrent Adaptor A reverse Barcode 154	TCACCGCTCGGATC/GATTGGTGGCGG/CTGAGTCGGAGACACGCA	CCGCCACCAATC
IonTorrent Adaptor A reverse Barcode 155	TCACCGCTCGGATC/GAATAACCTT/CTGAGTCGGAGACACGCA	AAGGTTATTC
IonTorrent Adaptor A reverse Barcode 156	TCACCGCTCGGATC/GACCAATCTCCA/CTGAGTCGGAGACACGCA	TGGAGATTGGTC
IonTorrent Adaptor A reverse Barcode_157	TCACCGCTCGGATC/GATTGATGGAGA/CTGAGTCGGAGACACGCA	TCTCCATCAATC
IonTorrent Adaptor A reverse Barcode 158	TCACCGCTCGGATC/GTGTTGGCTCCA/CTGAGTCGGAGACACGCA	TGGAGCCAACAC
IonTorrent Adaptor A reverse Barcode 159	TCACCGCTCGGATC/GAATCGATTAGA/CTGAGTCGGAGACACGCA	TCTAATCGATTC
IonTorrent Adaptor A reverse Barcode 160	TCACCGCTCGGATC/GTATTGGTGG/CTGAGTCGGAGACACGCA	CCACCAATAC
IonTorrent Adaptor A reverse Barcode_161	TCACCGCTCGGATC/GTCGAATCCAAG/CTGAGTCGGAGACACGCA	CTTGGATTCGAC
IonTorrent Adaptor A reverse Barcode 162	TCACCGCTCGGATC/GATAATCCAGAA/CTGAGTCGGAGACACGCA	TTCTGGATTATC
IonTorrent Adaptor A reverse Barcode 163	TCACCGCTCGGATC/GCCAGAAGAA/CTGAGTCGGAGACACGCA	TTCTTCTGGC
IonTorrent Adaptor A reverse Barcode 164	TCACCGCTCGGATC/GAGTCTCAGGA/CTGAGTCGGAGACACGCA	TCCTGAGACTC
IonTorrent Adaptor A reverse Barcode 165	TCACCGCTCGGATC/GTCTTGTTCCAG/CTGAGTCGGAGACACGCA	CTGGAACAAGAC
IonTorrent Adaptor A reverse Barcode 166	TCACCGCTCGGATC/GATTAAGCAAGA/CTGAGTCGGAGACACGCA	TCTTGCTTAATC
IonTorrent Adaptor A reverse Barcode 167	TCACCGCTCGGATC/GTCCAATTGGAG/CTGAGTCGGAGACACGCA	CTCCAATTGGAC
IonTorrent Adaptor A reverse Barcode 168	TCACCGCTCGGATC/GACCTTCCTTAG/CTGAGTCGGAGACACGCA	CTAAGGAAGGTC
IonTorrent Adaptor A reverse Barcode 169	TCACCGCTCGGATC/GAGGTGCCTTCA/CTGAGTCGGAGACACGCA	TGAAGGCACCTC
IonTorrent Adaptor A reverse Barcode_170	TCACCGCTCGGATC/GAACCGGATTGT/CTGAGTCGGAGACACGCA	ACAATCCGGTTC
IonTorrent Adaptor A reverse Barcode 171	TCACCGCTCGGATC/GTTCTGTAAGGA/CTGAGTCGGAGACACGCA	TCCTTACAGAAC
IonTorrent Adaptor A reverse Barcode 172	TCACCGCTCGGATC/GTTCGATTCA/CTGAGTCGGAGACACGCA	TGAATCGAAC
IonTorrent Adaptor A reverse Barcode 173	TCACCGCTCGGATC/GACGGCTTCAAG/CTGAGTCGGAGACACGCA	CTTGAAGCCGTC
IonTorrent Adaptor A reverse Barcode 174	TCACCGCTCGGATC/GATTGATCTCAA/CTGAGTCGGAGACACGCA	TTGAGATCAATC
IonTorrent Adaptor A reverse Barcode 175	TCACCGCTCGGATC/GTCGAATTGCTG/CTGAGTCGGAGACACGCA	CAGCAATTCGAC
IonTorrent Adaptor A reverse Barcode 176	TCACCGCTCGGATC/GATTAGCTTCG/CTGAGTCGGAGACACGCA	CGAAGCTAATC
IonTorrent Adaptor A reverse Barcode 177	TCACCGCTCGGATC/GTCAGCCTTAAG/CTGAGTCGGAGACACGCA	CTTAAGGCTGAC
IonTorrent Adaptor A reverse Barcode_178	TCACCGCTCGGATC/GTGGTTCTCCAG/CTGAGTCGGAGACACGCA	CTGGAGAACCAC
IonTorrent Adaptor A reverse Barcode 179	TCACCGCTCGGATC/GATTCCAAGTA/CTGAGTCGGAGACACGCA	TACTTGGAATC
IonTorrent Adaptor A reverse Barcode 180	TCACCGCTCGGATC/GAGGAGGCCTAG/CTGAGTCGGAGACACGCA	CTAGGCCTCCTC
IonTorrent Adaptor A reverse Barcode 181	TCACCGCTCGGATC/GTTGTTCTCGG/CTGAGTCGGAGACACGCA	CCGAGAACAAC
IonTorrent Adaptor A reverse Barcode 182	TCACCGCTCGGATC/GACGTCTTAA/CTGAGTCGGAGACACGCA	TTAAGACGTC
IonTorrent Adaptor A reverse Barcode_183	TCACCGCTCGGATC/GCGGATCTTAG/CTGAGTCGGAGACACGCA	CTAAGATCCGC
IonTorrent Adaptor A reverse Barcode 184	TCACCGCTCGGATC/GATGAAGCCA/CTGAGTCGGAGACACGCA	TGGCTTCATC
IonTorrent Adaptor A reverse Barcode 185	TCACCGCTCGGATC/GACAATTGTTCG/CTGAGTCGGAGACACGCA	CGAACAATTGTC
IonTorrent Adaptor A reverse Barcode 186	TCACCGCTCGGATC/GAACACCTTGAA/CTGAGTCGGAGACACGCA	TTCAAGGTGTTC
IonTorrent Adaptor A reverse Barcode 187	TCACCGCTCGGATC/GTGGTGGTTAAG/CTGAGTCGGAGACACGCA	CTTAACCACCAC
IonTorrent Adaptor A reverse Barcode 188	TCACCGCTCGGATC/GAACGGTCCGGA/CTGAGTCGGAGACACGCA	TCCGGACCGTTC
IonTorrent Adaptor A reverse Barcode 189	TCACCGCTCGGATC/GCATGCTCAAGG/CTGAGTCGGAGACACGCA	CCTTGAGCATGC
IonTorrent Adaptor A reverse Barcode 190	TCACCGCTCGGATC/GAATATCTAAGA/CTGAGTCGGAGACACGCA	TCTTAGATATTC
IonTorrent Adaptor A reverse Barcode 191	TCACCGCTCGGATC/GTAATTCAGG/CTGAGTCGGAGACACGCA	CCTGAATTAC
IonTorrent Adaptor A reverse Barcode 192	TCACCGCTCGGATC/GTTGGTTGGCTT/CTGAGTCGGAGACACGCA	AAGCCAACCAAC
Linker sequence	CGATCCGAGCGGTGA	
Linker-*pfama1*-forward	CGATCCGAGCGGTGA/CCATCAGGGAAATGTCCAGT	
Linker-*pfcsp*-forward	CGATCCGAGCGGTGA/ACAATCAAGGTAATGGACAAGG	
Linker-*pfk13*-forward	CGATCCGAGCGGTGA/TGCTGTATTGAATAATTTCT	
TrP1 Sequence	CCTCTCTATGGGCAGTCGGTGAT	
pfcsp-TrP1-reverse	CCTCTCTATGGGCAGTCGGTGAT/TTACGACATTAAACACACTGGAAC	
pfama1-TrP1-reverse	CCTCTCTATGGGCAGTCGGTGAT/TTTTTATCTTTGTCATCATAAACAGC	
pfk13-TrP1-reverse	CCTCTCTATGGGCAGTCGGTGAT/TTTATTATCAAAAGCAAC	
Final Forward	CCATCTCATCCCTGCGTGTCTCCGACTCAG	
Final Reverse	CCTCTCTATGGGCAGTCGGTGAT	

Table S1. Primer Sequences.

pf-csp

Position	Nucleotide	SNP
39	862	G862A
79	902	G902A
82	905	C905A
126	949	A949G
129	952	G952C
140	963	C963G
142	965	A965G
147	970	C970A
150	973	A973T
156	979	C979A
231	1054	A1054G
232	1055	A1055G
237	1060	C1060T
246	1069	G1069C
258	1081	G1081
259	1082	C1082
268	1091	T1091
270	1093	G1093
271	1094	A1094
279	1102	A1102
282	1105	T1105
283	1106	G1106
284	1107	T1107
285	1108	A1108
301	1124	C1124

pf-ama1

Position	Nucleotide	SNP
54	486	T486A
59	491	C491T
68	500	C500A
83	515	G515A
91	523	T523G
104	536	G536
106	538	G538A
108	540	T540G
113	545	C545
127	559	G559A
129	561	A561T
134	566	T566A
138	570	G570A
154	586	G586A
157	589	G589C
158	590	A590G
159	591	A591T
166	598	C598G
167	599	A599T
169	601	T601C
171	603	T603G
178	610	G610A
184	616	A616G
187	619	T619G
197	629	A629
204	636	T636
205	637	G637A
218	650	G650
242	674	T674A
266	698	A698T

Table S2. Alleles used to construct haplotypes.

ID	Date	Sex	Age	Location	RDT	Symptomatic
BC13	4/2014	male	<10	maraka	positive	
BC14	4/2014	male	<10	Ndivisi	positive	
BC15	4/2014	female	<10	Misikhu	positive	
BC16	4/2014	male	<10	Misikhu	positive	
BC17	4/2014	female	<10	Misikhu	positive	
BC18	4/2014	male	>10	Misikhu	positive	Yes
BC19	4/2014	male	<10	Misikhu	positive	
BC20	4/2014		<10	Sitikho	positive	
BC21	4/2014	male	<10	Misikhu	positive	
BC22	4/2014	female	>10	Webuye	positive	
BC23	4/2014	female	<10	Webuye	positive	Yes
BC24	4/2014	female	<10	Webuye	positive	
BC25	4/2014	female	>10	Sitikho	positive	
BC26	4/2014	male	>10	Sitikho	positive	Yes
BC27	5/2014	female	<10	Miendo	positive	
BC28	5/2014	male	<10	Miendo	positive	Yes
BC29	5/2014	male	<10	Miendo	positive	
BC30	5/2014	female	<10	Misikhu	positive	
BC31	5/2014	male	<10	Misikhu	positive	
BC32	5/2014	male	>10	Misikhu	positive	Yes
BC33	5/2014		<10	Sitikho	positive	
BC34	5/2014	female	<10	Sitikho	positive	Yes
BC35	9/2014	male	<10	Sitikho	positive	
BC36	9/2014	female	<10	Sitikho	positive	
BC37	9/2014	female	>10	Sitikho	positive	Yes
BC38	9/2014	male	<10	Sitikho	positive	Yes
BC39	9/2014	male	<10	Sitikho	positive	
BC40	5/2014	male	<10	Maraka	positive	
BC41	5/2014	male	<10	Sitikho	positive	
BC42	4/2014	male	<10	Yalusi	positive	
BC43	9/2014		<10	Muji	positive	
BC44	9/2014	male	<10	Sitikho	positive	
BC45	5/2014	male	<10	Matulo	positive	
BC46	5/2014	male	<10	Matulo	positive	Yes
BC47	5/2014	male	<10	Matulo	positive	Yes
BC48	9/2014	female	<10	Sitikho	positive	
BC49	9/2014	female	>10	Sitikho	negative	
BC50	5/2014	male	<10	Muchi	positive	
BC51	5/2014	male	<10	Muchi	positive	Yes
BC52	5/2014	male	<10	Webuye	positive	
BC53		female	<10	Makemo	positive	
BC54	4/2014	female	<10	matulo	positive	
BC55	9/2014		<10	Maraka	positive	
BC56	9/2014	female	>10	Maraka	positive	
BC57	9/2014	female	<10	matulo	positive	
BC58		female	<10	Maraka	positive	
BC59	5/2014		<10	Webuye	positive	
BC60			<10	Muji	positive	
BC61	5/2014	female	<10	Webuye	positive	
BC62	5/2014	male	<10	Miendo	positive	
BC63	5/2014	male	<10	Misikhu	positive	
BC64	5/2014	male	<10	Maraka	positive	
BC65		female	<10	Maraka	positive	
BC66	5/2014	female	>10	Muchi	positive	Yes
BC67	5/2014	female	<10	Muchi	positive	Yes
BC68	5/2014	female	<10	Muchi	positive	Yes
BC69	5/2014	female	<10	Muchi	positive	Yes
BC70	5/2014	male	<10	Ndivisi	positive	
BC71	5/2014	female	<10	Ndivisi	positive	Yes
BC72	5/2014	female	<10	Webuye	positive	
BC73	5/2014	male	<10	Sitikho	positive	
BC74	5/2014	male	>10	Misikhu	positive	Yes
BC75		male	>10	Sitikho	positive	Yes
BC76		female	<10	Sitikho	positive	
BC77		female	>10	Sitikho	positive	
BC78		female	>10	Sitikho	positive	
BC79		female	<10	Matisi	positive	
BC80		female	>10	Matisi	positive	
BC81	7/2014	male	<10	Webuye	positive	
BC82	7/2014		<10	Webuye	positive	
BC83	7/2014	female	>10	Webuye	positive	

BC84	7/2014	male	<10	Maraka	positive	
BC85	7/2014	female	<10	Sitikho	positive	
BC86	7/2014	female	<10	Sitikho	positive	Yes
BC87	7/2014	female	<10	Misikhu	positive	
BC88	7/2014	male	<10	Matulo	positive	
BC89	7/2014	female	<10	Sitikho	positive	
BC90	7/2014	female	<10	Sitikho	positive	Yes
BC91	7/2014	female	>10	Sitikho	positive	Yes
BC92	7/2014	female	>10	Sitikho	positive	Yes
BC93		male	<10	Misikhu	positive	Yes
BC94	7/2014	male	<10	Muchi	Positive	
BC95	7/2014		<10	Muchi	Positive	
BC96	7/2014	female	<10	Sitikho	positive	
BC97	7/2014	male	<10	Sitikho	positive	yes
BC98	7/2014	female	>10	Sitikho	positive	yes
BC99	7/2014	female	<10	Chetambe	positive	
BC100	9/2014		<10	Sitikho	positive	
BC101			<10	Webuye	positive	
BC102		female	>10	Webuye	positive	
BC103		female	>10	Webuye	positive	Yes
BC104		female	<10	Webuye	positive	
BC105	9/2014	male	<10	Webuye	positive	
BC106		male	<10	Maraka	positive	Yes
BC107		female	<10	Maraka	positive	Yes
BC108		male	>10	Maraka	positive	Yes
BC109		female	>10	Maraka	positive	Yes
BC110	9/2014	male	<10	Matulo	positive	
BC111		male	>10	Miendo	positive	Yes
BC112	9/2014	male	>10	Sitikho	positive	
BC113	7/2014	female	>10	Webuye	positive	Yes
BC114	7/2014	male	<10	Webuye	positive	Yes
BC115		female	<10	Chetambe	positive	Yes
BC116	7/2014	male	>10	Sitikho	positive	
BC117		female	>10	Mihuu	positive	Yes
BC118		male	<10	Mihuu	positive	Yes
BC119	9/2014	male	>10	Sitikho	positive	
BC120	9/2014	female	<10	Matulo	positive	
BC121	9/2014	female	>10	Matulo	positive	Yes
BC122	9/2014	male	<10	Matulo	positive	Yes
BC123		female	>10	Maraka	positive	Yes
BC124		female	<10	Maraka	positive	Yes
BC125		male	<10	Maraka	positive	Yes
BC126	9/2014	female	<10	Sitikho	positive	Yes
BC127	8/2014	male	>10	Misikhu	positive	
BC128	8/2014	male	>10	Sitikho	positive	
BC129	8/2014	male	<10	Sitikho	positive	
BC130	8/2014	female	<10	Sitikho	positive	Yes
BC131	8/2014	male	<10	Sitikho	positive	Yes
BC132	8/2014	male	>10	Sitikho	positive	
BC133	8/2014		<10	Sitikho	positive	Yes
BC134	8/2014	female	>10	Sitikho	positive	
BC135	7/2014	male	<10	Misikhu	positive	Yes
BC136		female	>10	Mihuu	positive	Yes
BC137		female	>10	Mihuu	positive	Yes
BC138		female	>10	Mihuu	positive	
BC139		female	<10	Misikhu	positive	
BC140		female	<10	Misikhu	positive	
BC141	7/2014	female	>10	Sitikho	positive	
BC142	7/2014	female	>10	Sitikho	positive	
BC143	7/2014	male	>10	Sitikho	positive	
BC144	8/2014	male	<10	Misikhu	positive	Yes
BC145	8/2014	female	>10	Misikhu	positive	Yes
BC146	7/2014	female	<10	Sitikho	positive	Yes
BC147	7/2014	male	<10	Sitikho	positive	Yes
BC148	7/2014	male	>10	Sitikho	positive	Yes
BC149		female	>10	Misikhu	positive	Yes
BC150		female	<10	Misikhu	positive	Yes
BC151		female	<10	Misikhu	positive	Yes
BC152	7/2014	male	>10	Misikhu	positive	
BC153	7/2014	female	<10	Misikhu	positive	
BC154	7/2014	female	<10	Misikhu	positive	
BC155	7/2014	female	>10	Matulo	positive	Yes
BC156	7/2014	female	>10	Matulo	positive	
BC157	7/2014	male	>10	Matulo	positive	

BC158	8/2014	female	>10	Sitikho	positive	Yes
BC159	8/2014	female	<10	Maraka	positive	Yes
BC160	8/2014	male	>10	Maraka	positive	Yes
BC161	8/2014	female	>10	Misikhu	positive	Yes
BC162	8/2014	male	>10	Misikhu	positive	Yes
BC163	8/2014	male	<10	Webuye	positive	Yes
BC164	8/2014	female	>10	Sitikho	negative	
BC165	8/2014	female	>10	Sitikho	positive	Yes
BC166	8/2014	female	>10	Sitikho	positive	Yes
BC167	7/2014	female	>10	Sitikho	positive	Yes
BC168	7/2014	male	<10	Sitikho	Positive	
BC169	7/2014	male	<10	Sitikho	positive	Yes
BC170		male	>10	Sitikho	positive	
BC171		male	>10	Sitikho	positive	
BC172		female	>10	Maraka	positive	Yes
BC173		female	<10	Maraka	positive	Yes
BC174		female	<10	Webuye	positive	Yes
BC175		male	>10	Sitikho	positive	Yes
BC176		female	>10	Sitikho	positive	Yes
BC177		male	>10	Sitikho	positive	
BC178	7/2014	male	>10	Sitikho	positive	Yes
BC179	7/2014	female	>10	Sitikho	positive	Yes
BC180	8/2014	female	>10	Matulo	positive	Yes
BC181	8/2014	male	>10	Matulo	positive	
BC182	8/2014	female	>10	Matulo	positive	Yes
BC183	8/2014		<10	Matulo	positive	
BC184	8/2014	female	<10	Matulo	positive	Yes
BC185	7/2014		<10	Muchi	positive	
BC186	7/2014	female	>10	Muchi	negative	
BC187	7/2014	female	>10	Muchi	positive	Yes
BC188	7/2014	female	<10	Muchi	positive	

Table S3. Clinical and demographic information all patients. ID indicates patient identifier by barcode number. Date refers to the date of collection of blood sample. RDT status indicates whether they tested positively by the *P. falciparum* antigen HRP2 (Standard Diagnostics SD Bioline Malaria Ag Pf (HRPII))

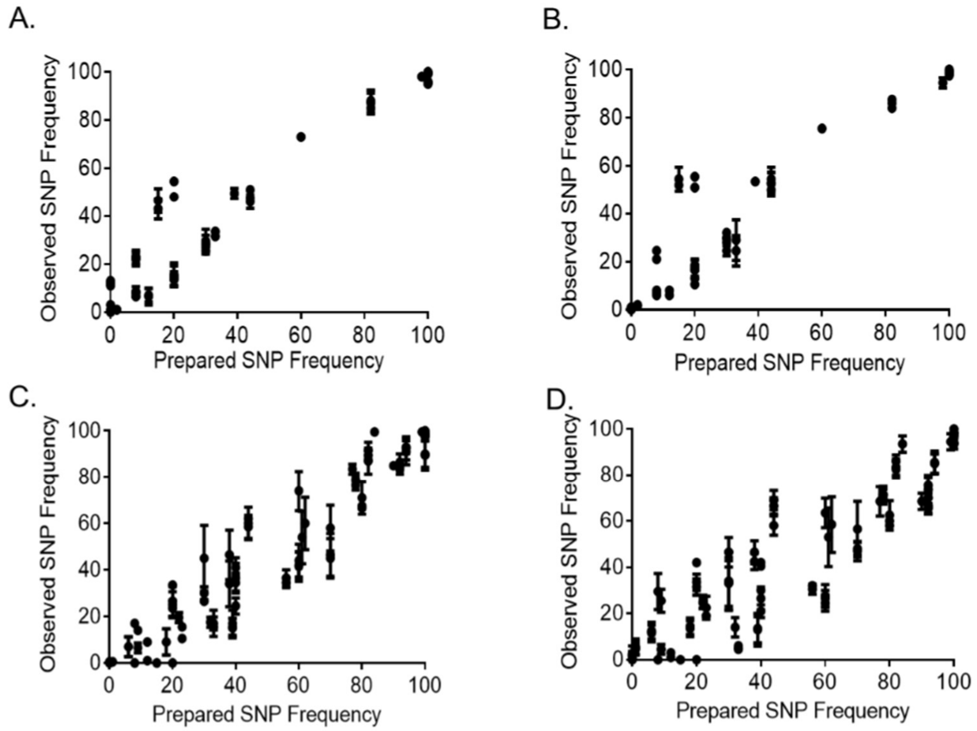

Figure S1. Experimental replicates of IonTorrent analysis. A. IonTorrent replicate 1 for *pf-csp*. B. IonTorrent replicate 2 for *pf-csp*. C. IonTorrent replicate 1 for *pf-ama1*. D. IonTorrent replicate 2 for *pf-ama1*.

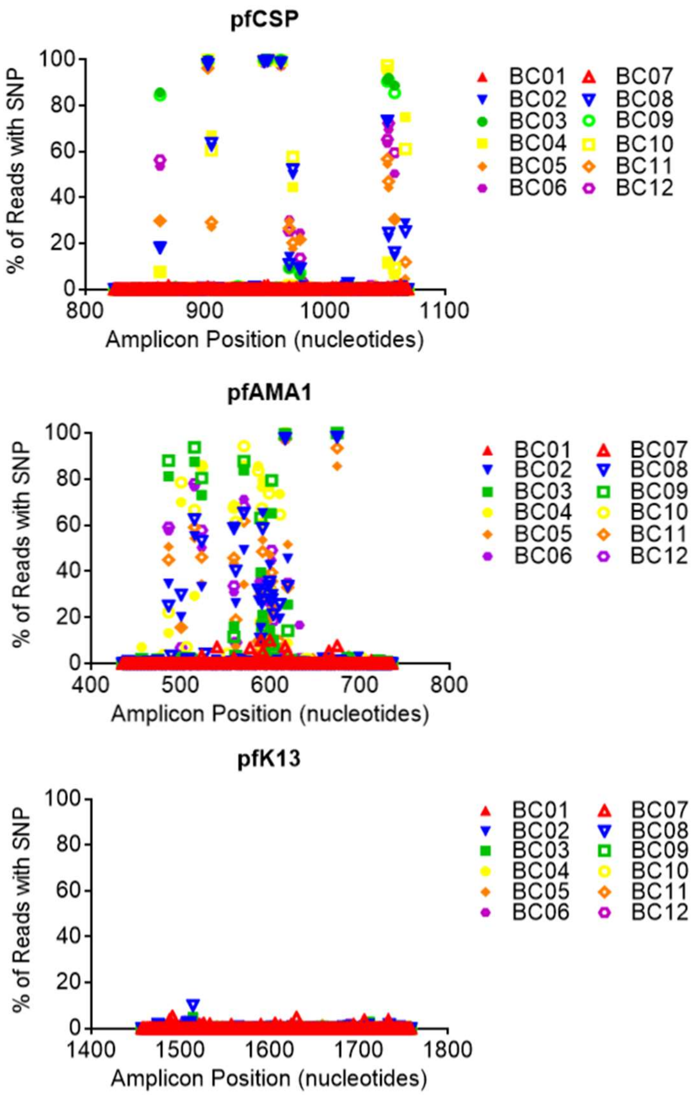

Figure S2. Positions and frequency of SNPs in mock-infected controls for each gene target.

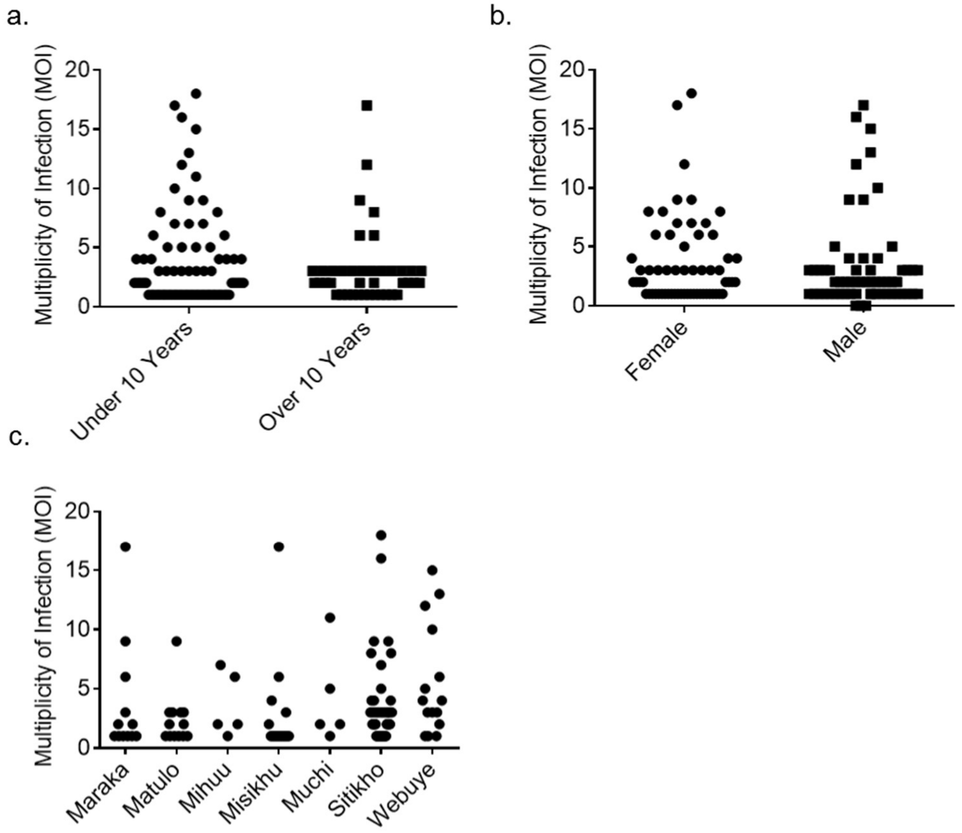

Figure S3. Multiplicity of infection (MOI) stratified by clinical and demographic features. a. Sorted by age. b. Sorted by gender. c. Sorted by location

a

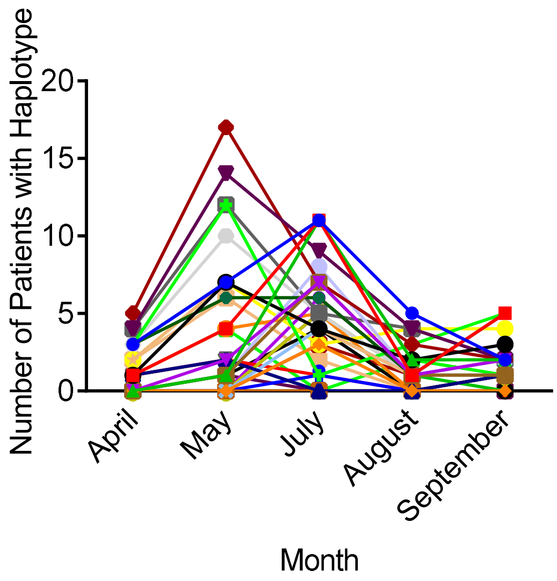

b.

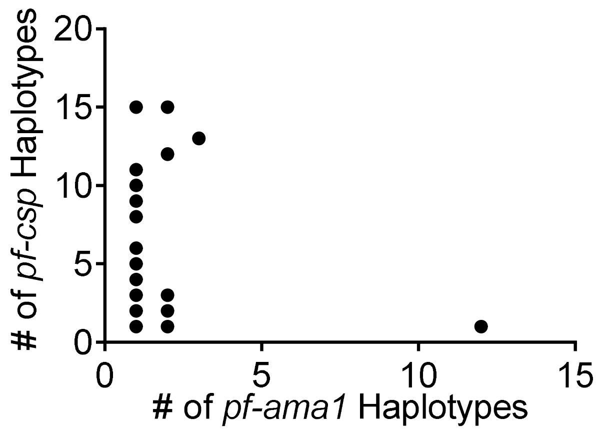

Figure S4. Analysis of Haplotypes in Field Collected Samples. a. Seasonal trends of each of the *pf-csp* haplotypes over several months. b. Concordance of number of *pf-ama1* and *pf-csp* haplotypes.
